# Clinical Case Summary and Presentation: A Pilot Study to Address the Gap and Improve Medical Students’ Performance in Bedside Teaching

**DOI:** 10.7759/cureus.87539

**Published:** 2025-07-08

**Authors:** Asmaa Omran, Nichola Philp, Michael J Otorkpa, Alan Kirk

**Affiliations:** 1 Cardiothoracic Surgery, Golden Jubilee National Hospital, Glasgow, GBR; 2 School of Medicine, University of Glasgow, Glasgow, GBR; 3 Medical Education, University of Dundee, Dundee, GBR; 4 Thoracic Surgery, Golden Jubilee National Hospital, Glasgow, GBR

**Keywords:** bedside teaching, cardiac surgery, case summary and presentation, clinical education, communication skills, non-technical skills teaching, undergraduate medical students

## Abstract

Introduction

Bedside teaching is a well-known clinical teaching method that provides medical students with a real-life experience and patient interaction. As future doctors, medical students need to learn not only how to take a history and perform an examination but also how to present their findings. Providing a summary of a clinical case and presenting it back is an important non-technical skill that is often overlooked during bedside teaching.

Objective

The aim of this prospective pilot study was to evaluate the outcome of teaching undergraduate medical students how to provide a verbal case summary and presentation.

Methods

A total of 45 fourth-year medical students from the University of Glasgow (UOG) completed a pre-teaching survey in two groups: 25 students (group A) attended small group bedside teaching in a cardiac surgery ward only, while 20 students (group B) attended a large group tutorial prior to the bedside session. During the bedside sessions, each student undertook a history and examination and then provided a clinical summary of their encounter to the supervisor. The student and the supervisor then completed a post-teaching assessment.

Results

Of the 45 students, 33 (73%) were “somewhat confident” with their presentation skills; only 28 (62%) had received previous teaching about clinical case summary, while 23 (51%) did not know which information to include or which to omit when summarising. The supervisor’s assessment demonstrated that 16 (84%) students’ presentations out of 19 in group A were “very good” or “excellent” in terms of being in chronological order, 11 (58%) in using medical terms, 10 (53%) in being concise, 15 (79%) in being clear, and 13 (68%) in being comprehensive. These numbers increased in group B to 18 (90%) out of 20, 16 (80%), 13 (65%), 16 (80%), and 15 (75%), respectively, with the greatest improvement being increased use of medical terms.

Using the students' self-assessment, 20 (80%) out of 25 students in group A evaluated their case summary and presentation as being in chronological order, 20 (80%) used medical terms, 15 (60%) were concise, 15 (60%) were clear, and 12 (48%) were comprehensive. In group B, the evaluation of these parameters changed to 15 (75%) out of 20, 15 (75%), eight (40%), 11 (55%) and 13 (65%), respectively. The greatest reduction occurred in the conciseness of the presentations, while there was an increase in the comprehensiveness parameter.

All students from group B evaluated both the tutorial and the bedside session positively across all parameters; 14 (65%) rated their confidence in providing a case summary and presentation after the teaching session between 4 and 5, while 18 (90%) expected their verbal communication skills to improve on a scale of 4-5.

Conclusion

Group B showed improved performance in all parameters according to the supervisor’s assessment, whereas group A students rated their own performance higher than that of group B. More focused and structured teaching about clinical case summary and presentation is required to address the gap in undergraduate medical education.

## Introduction

Bedside teaching is one of the most important sessions medical students attend during their clinical placement [1]. Each session involves up to five students supervised and guided by two clinical teaching fellows (CTFs) (A.O., M.O., or N.P.). The students take a history and perform a cardiovascular examination on a patient admitted to a cardiac surgery ward. The students then present their case to their supervisor, who provides feedback in a safe environment away from the patient. Informed verbal consent was obtained from patients before the session, and no patients’ data were included in this study.

Providing a verbal presentation of a consultation with a patient is a crucial non-technical skill that is often overlooked during teaching, learning, and assessment in undergraduate medical education. Students perceive the presentation as an ordered, structured, rule-based data storage activity, whereas teachers perceive a presentation as a flexible means of intra-professional communication to construct the details of a case into a diagnostic or therapeutic plan [2-4]. These different perceptions constitute a gap between what is relevant to the medical student and what is relevant to the teacher in the provided presentation [2-4]. This pilot study aimed to evaluate the outcome of teaching medical students how to provide a verbal clinical case summary and presentation.

## Materials and methods

Fourth-year medical students from the University of Glasgow (UOG), Scotland, attend Golden Jubilee University National Hospital (GJNH), a tertiary care centre, for their cardiology and cardiothoracic clinical placement; 23 to 25 students attend the hospital for two and a half weeks each block.

Two consecutive groups of fourth-year medical students between May and June 2024 participated in the study. Students were asked to fill out a pre-teaching survey (Appendix A) to evaluate their confidence in providing a verbal summary of their consultation with a patient; whether they had received any prior teaching about providing a case summary and presentation; and the reason or reasons that they think case presentations are challenging. Survey results from group A were used to guide the design of the tutorial session.

The second group of students (group B) attended a tutorial teaching session (Appendix B) on how to deliver a verbal case summary and presentation. The primary learning outcomes [5] remained unaffected as both groups continued to attend the main bedside teaching session.

The Health Research Authority decision tool indicated that ethical approval was not required, and informed verbal consent was obtained from students.

After the bedside teaching, students in both groups summarised and verbally presented their case back to their supervising CTF. Each student’s summary and presentation were assessed by the CTF using a five-point Likert scale questionnaire developed by the assessors (Appendix C), assessing the student’s provided summary: whether it was concise, clear, in chronological order, using medical terms instead of the patient’s own words, and whether it was comprehensive, including the disease, complications, and treatment (when applicable). The assessors gave a rating from one to five, where one is unsatisfactory, two is below average, three is average, four is very good, and five is excellent.

In addition to the supervisor’s assessment, each student was asked to fill out a self-assessment questionnaire (Appendix D) using the same criteria assessed by the supervisor. Students were also asked whether they encountered any difficulties understanding what the patient was talking about, which may have affected their summary. This question served to reflect the individual variations between different patients and the complexity of their clinical problems. Finally, questionnaires from both groups were compared.

Students from group B were also asked to fill in a post-teaching survey (Appendix E) to evaluate the tutorial teaching session using a five-point Likert questionnaire. Students were asked to give a rating from 1 to 5 (5 being the best), evaluating how useful the session was, how relevant it was to their learning needs, how applicable it was to their practice at the bedside, and how useful the format was. They were also asked to rate their confidence after the session in delivering a verbal summary of a consultation and whether they expected their communication skills to improve. In addition, students were asked to elaborate on what they found useful and what could be improved.

An online follow-up survey (Appendix F) was emailed to students from group B one month after the teaching session. The survey used a five-point Likert scale, where five is the highest rate, asking students to evaluate their recollection of the teaching session and whether they applied it in their following clinical placement.

## Results

In total, 45 fourth-year medical students participated in this study. In group A, there were 25 students, with an age range of 21-29 years (median 22 years): 20 students (80%) identified as female and 5 (20%) identified as male, including six international students and three second-degree undergraduates. In group B, there were 20 students, with an age range of 21-27 years (median 23 years): 11 students (55%) identified as female and 9 (45%) identified as male, including four international students and four second-degree undergraduates. Data were collected and analysed using simple statistics in Microsoft Forms and Microsoft Excel (Microsoft Corporation, Redmond, WA, USA).

Pre-teaching survey

Overall, 33 (73%) students in total were “somewhat confident” with their summary and presentation skills (Table 1), 10 students were “not confident”, two chose “not sure”, and no one chose “very confident”. Group A's average confidence on the Likert scale was 2.72, while group B's average was 2.25.

**Table 1:** Students' confidence pre-teaching

How confident?	Group A (%)	Group B (%)	Total (%)
Not confident	3 (12)	7 (35)	10 (22.2)
Not sure	1 (4)	1 (5)	2 (4.4)
Somewhat confident	21 (84)	12 (60)	33 (73.3)
Very confident	0	0	0
Total	25	20	45

Only 28 (62%) had received previous teaching (Table 2) about clinical case summary, with one student elaborating "only information" and another student mentioning SBAR (Situation, Background, Assessment, Recommendation) [6]; 10 students did not receive previous teaching, and seven were “not sure”.

**Table 2:** Number of students who received previous teaching about case summary and presentation

Previous teaching	Group A (%)	Group B (%)	Total (%)
No	3 (12)	7 (35)	10 (22.2)
Not sure	5 (20)	2 (10)	7 (15.5)
Yes	17 (68)	11 (55)	28 (62.2)
Total	25	20	45

**Figure 1:**
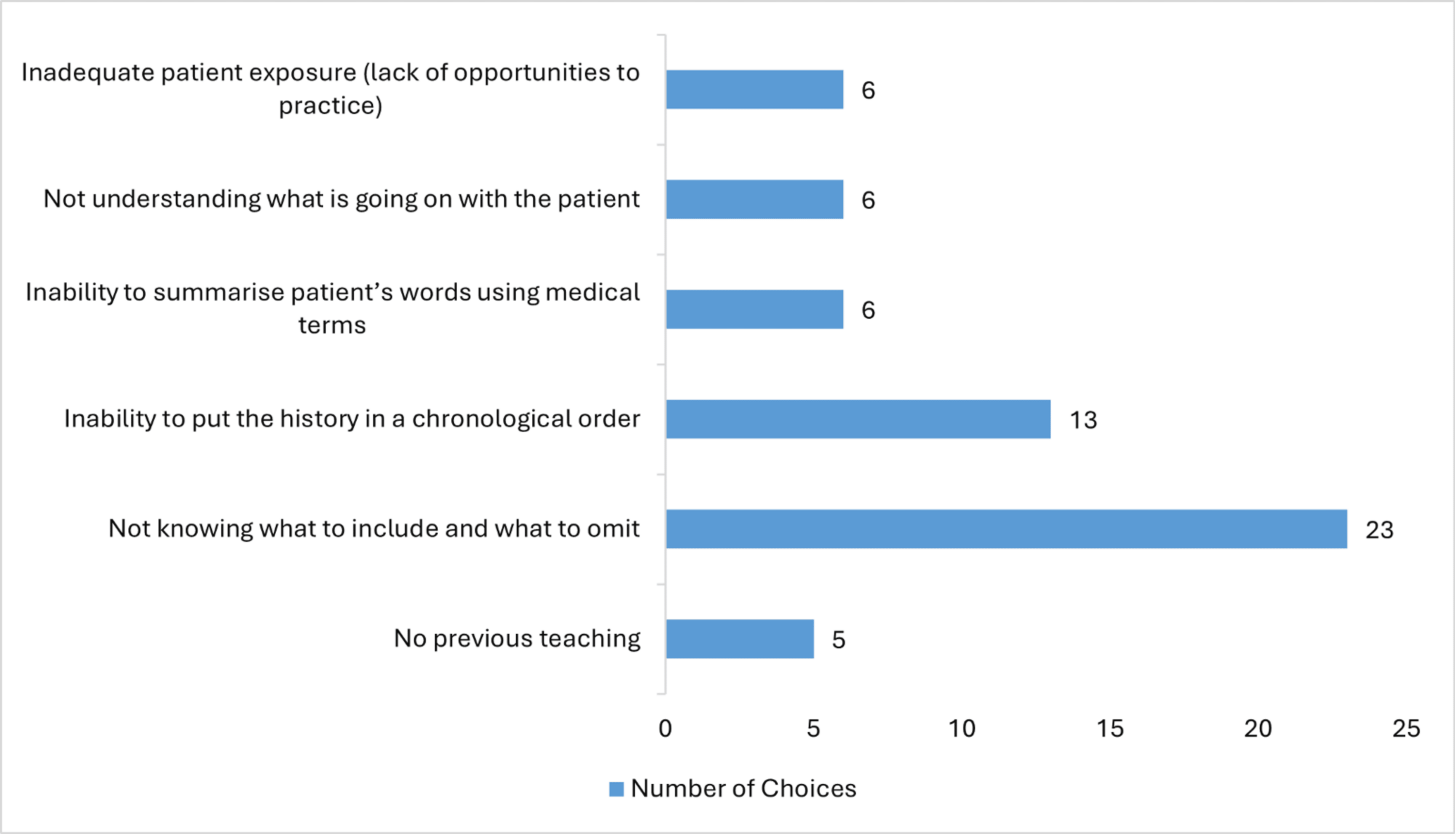
Reasons for challenging case presentations

Reasons contributing to challenges in providing a verbal summary (Figure 1) varied between no previous teaching, chosen by five students; inability to summarise the patient’s words using medical terms; not understanding what is going on with the patient; inadequate patient exposure; or lack of opportunities to practise, each chosen by six students. The most common chosen reasons were not knowing which information to include and which to omit when summarising a case, chosen by 23 (51%) students, and inability to put the history in a chronological order, chosen by 13 (28.8%) students. Students could select multiple answers for this question.

Supervisor's assessment

Initially, six students from group A were assessed using yes or no questions; however, assessors found these questions difficult to answer and not reliable for assessment. Therefore, the supervisor’s assessment was changed from yes or no questions to a five-point Likert scale questionnaire assessing the same parameters. The six initial assessments were excluded from the results.

**Figure 2:**
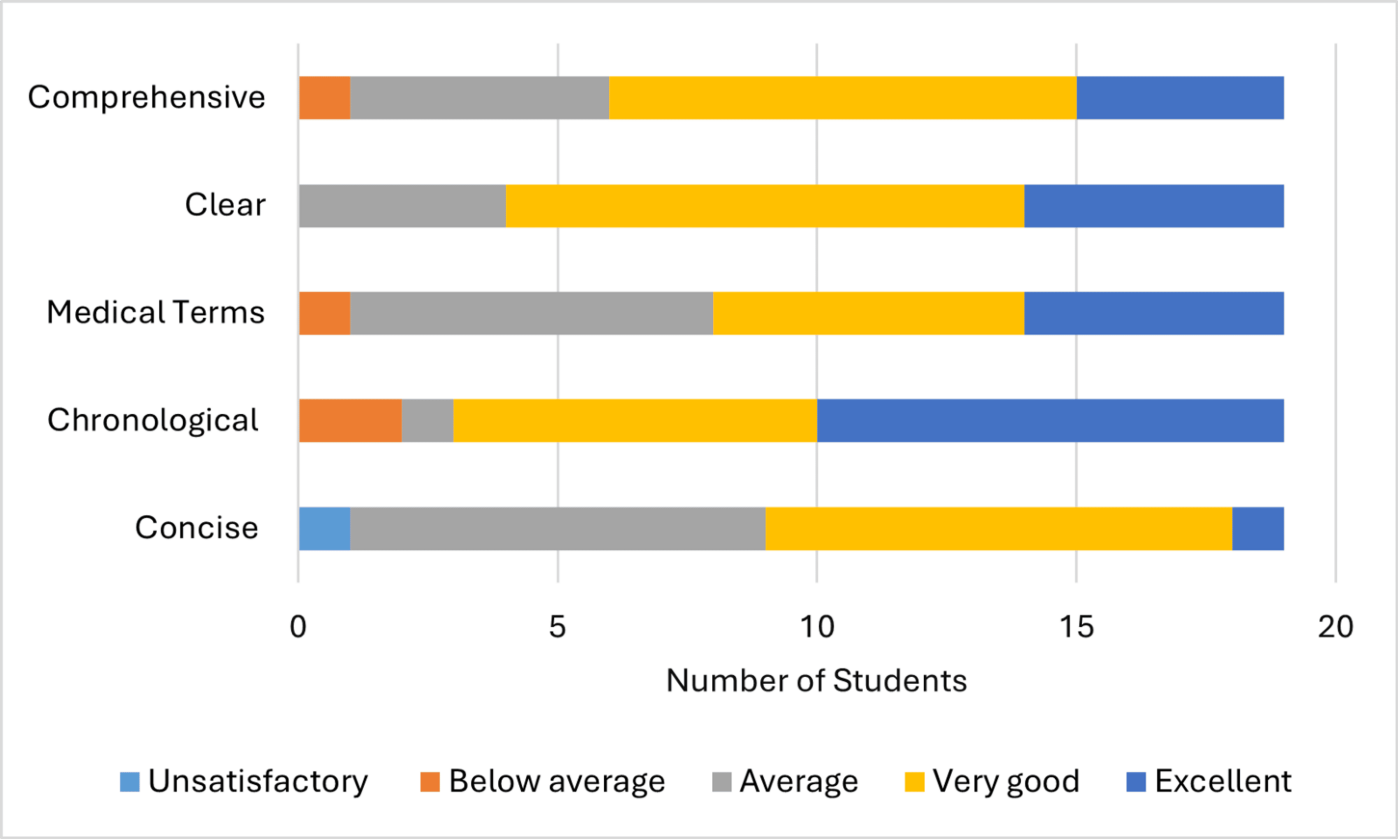
Supervisor's assessment (group A)

**Figure 3:**
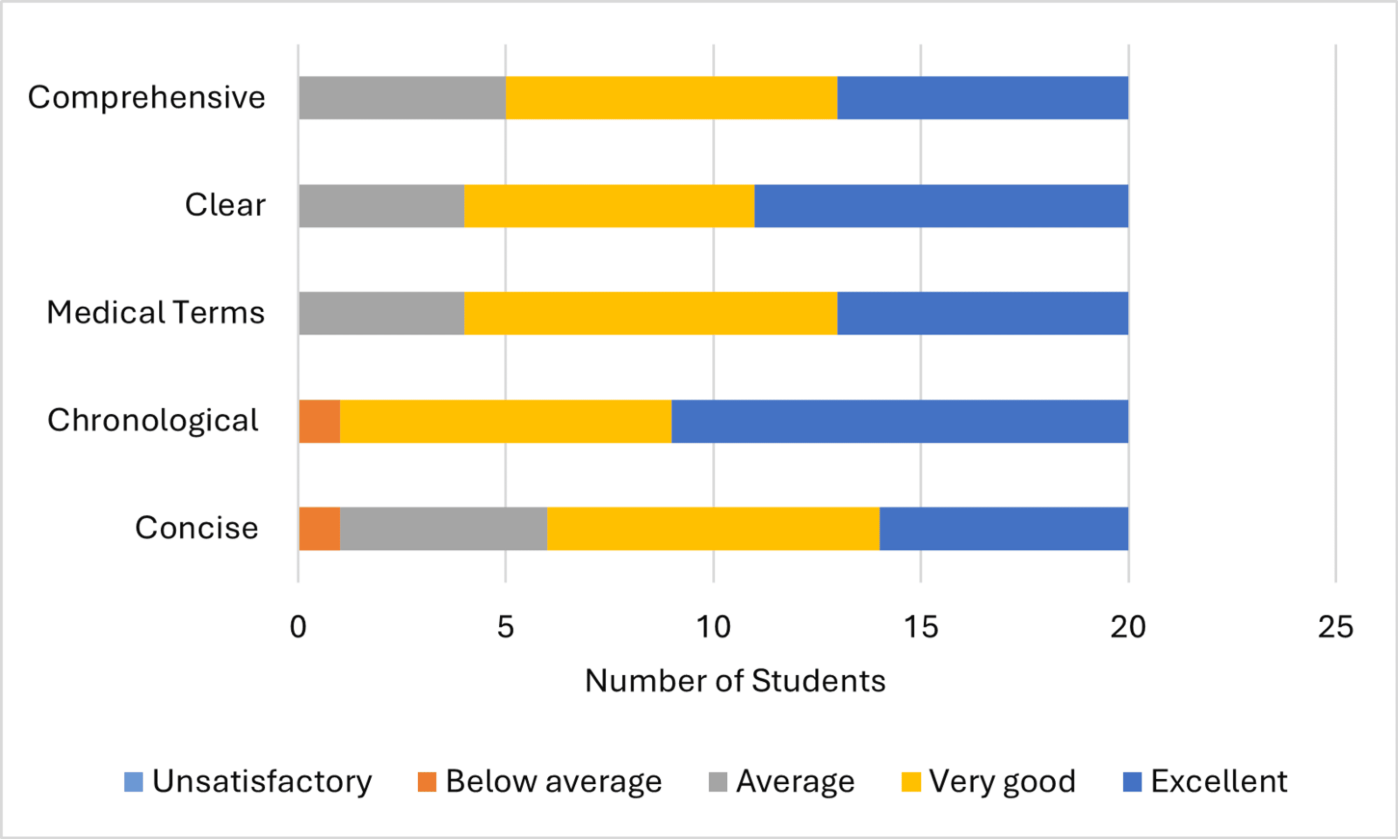
Supervisor's assessment (group B)

A total of 16 (84%) students’ presentations out of 19 students in group A (Figure 2) were "very good" or "excellent" in terms of being in chronological order, 11 (58%) in using medical terms, 10 (53%) being concise, 15 (79%) being clear, and 13 (68%) being comprehensive. These numbers increased in group B (Figure 3) to 18 (90%) out of 20, 16 (80%), 13 (65%), 16 (80%), and 15 (75%), respectively, with the highest improvement being increased use of medical terms.

Students' self-assessment

**Figure 4:**
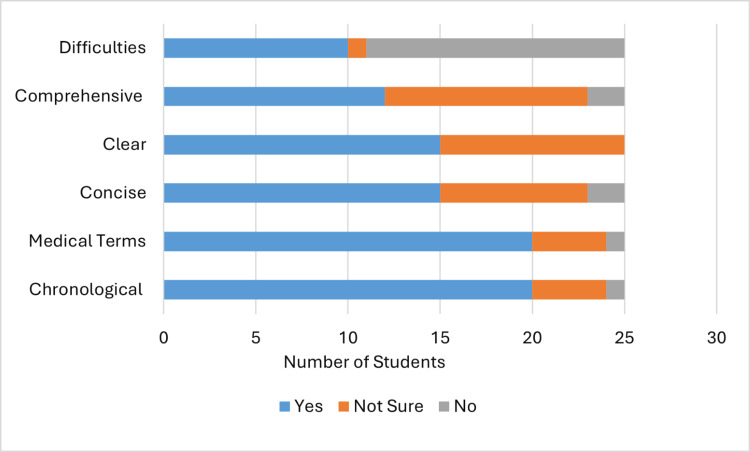
Students' self-assessment (group A)

**Figure 5:**
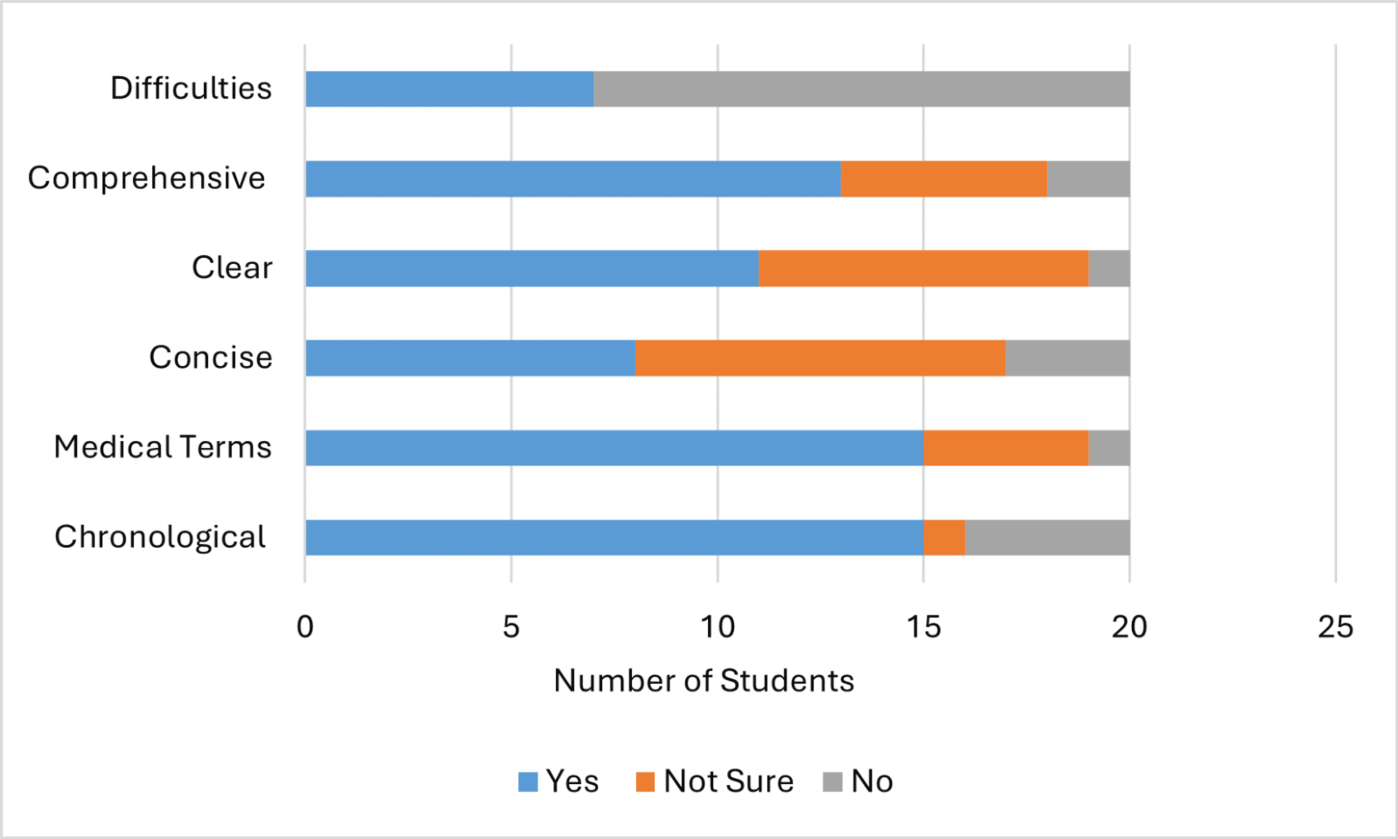
Students' self-assessment (group B)

Overall, 20 (80%) of 25 students in group A (Figure 4) evaluated their case summary and presentation as being in chronological order, 20 (80%) used medical terms, 15 (60%) were concise, 15 (60%) were clear, 12 (48%) were comprehensive, and 10 (40%) encountered difficulties understanding what the patient was talking about, which, in turn, negatively affected their summary (Table 3). These parameters changed in group B (Figure 5) to 15 (75%) out of 20 presentations being in chronological order, 15 (75%) in medical terms, eight (40%) concise, 11 (55%) clear, and 13 (65%) comprehensive, while only seven (35%) encountered difficulties (Table 3). The lowest reduction was in the presentations being concise, with an increase in the comprehensiveness parameter.

**Table 3:** Students' elaborations on the encountered difficulties (students' Free-Text Feedback)

Students’ elaborations on the encountered difficulties
Talking quickly.
Patient was unsure of exact timeline of events.
Tricky case to summarise as under what surgery was (assessor’s comment).
Regarding valve pathologies.
Sometimes mumbled.
Sometimes (mostly fine).
Visual changes.
Patient was uncertain, difficult to establish temporality of symptoms.
Had trouble understanding chronological order of things.

Post-teaching survey 

**Figure 6:**
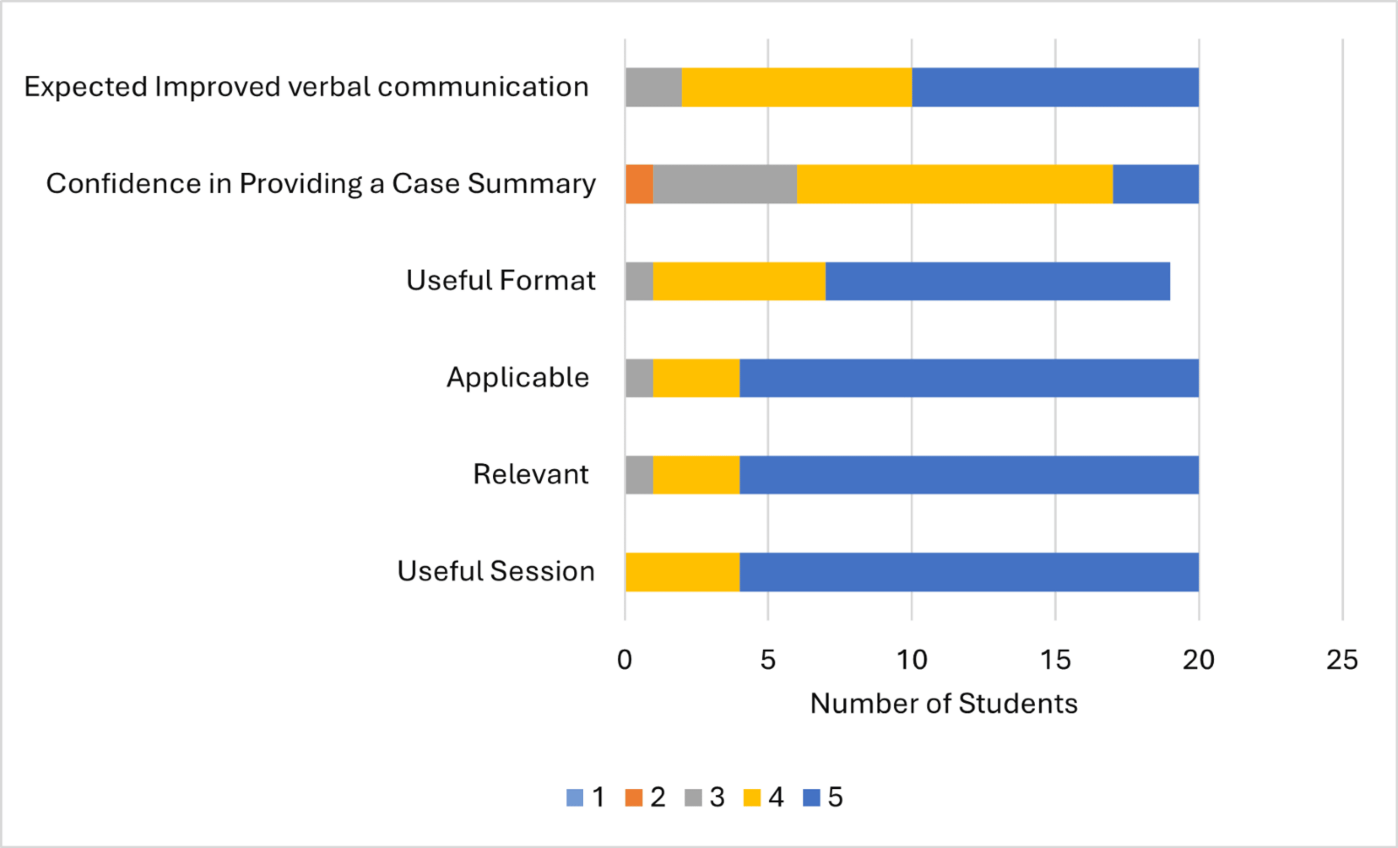
Post-teaching survey Students gave a rating from 1 to 5, with 5 being the best.

All the students from group B evaluated the tutorial session positively (Figure 6). They found it useful, with an average score of 4.8 on the Likert scale; relevant (average 4.75); applicable (average 4.75); and in a useful format (average 4.58), although one student did not answer this question.

Eleven (55%) students evaluated their confidence after the session with a score of 4 on the Likert scale, five (25%) gave a score of 3, three (15%) gave a score of 5, and one (5%) gave a score of 2, while no one chose a score of 1. There was an increase in average confidence to 3.8 from 2.25 pre-teaching. Ten (50%) students evaluated the improvement in their communication skills at a score of 5, eight (40%) gave a score of 4, two (10%) gave a score of 3, and no one chose a score of 2 or 1 (average 4.4). Students’ free-text feedback on the session is included in Table 4.

**Table 4:** Post-teaching survey (students’ free-text feedback) Mini-CEX (Mini Clinical Evaluation Exercise) is used as a formative assessment during bedside teaching.

What was useful?	What can be improved?	Any comments?
Discussed cases afterwards.	Bedside teaching was a bit after the teaching, so I forgot a bit.	Thank you.
Very in-depth discussion on findings.	N/A	Thank you.
It is useful to discuss cases after + practice summary.	Timing.	Thank you for giving us feedback and for teaching us today.
Being able to do a full history, examination + presentation on one patient.	Maybe more clear participants.	Very useful learning and discussion points.
Mini-CEX, full history + examination.	Maybe discussing immediately after taking a history because I forgot my patient’s details.	Very useful, thank you.
Practising history, examination, and summaries.	None.	-
Good practical tips on summarising.	I think the teaching session went well.	-
Helpful to summarise and run through the cases after.	More bedside teaching, if possible, please.	-
A chance to practise.	Nothing.	-
I found it useful to perform the examination and take the history for one patient, as usually for bedside teaching we always have to split the history and examination between at least two people, so I found today’s teaching format to be more useful. It was also useful to present the case at the end, as we do not practise presenting enough, so it helps. I also liked that we received feedback and were able to discuss the case, and it always helps my learning when we are asked about pathology.	Another bedside session.	-
Chatting about the cases afterwards, getting to do history and examination on my own.	Example?	-
Opportunity for Mini-CEX, great to assess patients with examination signs and pathology.	-	-
Good for handover.	-	-
Being able to do both history + examination, good to practise summary.	-	-
Talking through and feedback at the end.	-	-
Relevant.	-	-

Follow-up survey

Only six (30%) students filled out the online follow-up survey; five students rated their recollection of teaching between 4 and 5, and four rated their application of the teaching session in their subsequent clinical placement between 4 and 5. Students were asked to elaborate on the reasons for low ratings (Table 5).

**Table 5:** Follow-up survey (students’ free-text feedback)

If you rated your application of teaching low, what do you think was the reason?
I forgot to apply it.
I did not have a chance to verbally present a case.
I did not have a chance to verbally present a case.
Can be tricky to apply to complex cases with lots of detail.

## Discussion

Teaching case summary and presentation

Communication skills represent a core component of healthcare and hence of clinical education. While teaching communication skills between medical students as future doctors and patients has been extensively researched [7], there is limited research about teaching the doctor-to-doctor communication side. Some tools have been suggested to provide a framework for communication between clinical educators and medical students. The One-Minute Preceptor involves the following five steps: get a commitment, probe for supporting evidence, teach general rules, reinforce what was done right, and correct mistakes. The Summarise, Narrow, Analyse, Probe, Plan, Select (SNAPPS) tool includes the following six steps: summarise briefly the history and findings, narrow the differential to two or three relevant possibilities, analyse the differential by comparing and contrasting the possibilities, probe the preceptor by asking questions; plan management for the patient's medical issues, and select a case-related issue for self-directed learning. However, these tools do not provide details for students about how to summarise [8,9].

Another common tool that is usually used for handover (communication of the relevant patient’s information to another professional to transfer responsibility and accountability for some or all aspects of care for the patient) is SBAR. SBAR provides a structured means of communication between different professions in the healthcare setting, and while the structure is useful and important, limited details on how to summarise a case are provided within this structure [6]. Therefore, students end up learning how to provide a summary and presentation by observation, experience, trial and error, and without explicit teaching, which may lead to delayed acquisition of important communication skills [3].

Assessment of a verbal presentation

Assessment of a verbal case summary and presentation is a complex process not only due to the wide variety of patients and clinical problems but also due to a direct relation between clinical reasoning and delivering an effective verbal presentation [10,11]. Our supervisor’s assessment (Appendix C) addressed mainly five aspects of a presentation, which were targeted by the tutorial session. The main focus of our assessment was on the presentation of the history part of a clinical consultation, with limited attention to the examination findings, while Lewin et al. provided a more detailed, reliable, and comprehensive tool for presentation assessment in their study in 2013 [11].

The RIME (Reporter, Interpreter, Manager, Educator) scheme is another framework that was suggested by Pangaro [4,12]. It describes four developmental stages of medical trainees: (a) Reporter, who gathers and communicates clinical information, (b) interpreter, who analyses and interprets data to make clinical decisions, (c) manager, who develops and implements patient management plans, and (d) educator, who teaches others and contributes to the learning environment. The framework serves as a guide for educators on providing formative assessment and suggests the use of standardised vocabulary. In addition, it integrates feedback into the process of assessment. A multifaceted intervention by Green et al. managed to improve medical students’ presentation skills [13]; however, unfortunately, limited details have been provided about the assessment tool that was used to evaluate the outcome.

Clinical reasoning and case presentation

Clinical reasoning is a complex umbrella term that can be defined and used in several ways. One author defined clinical reasoning as the physician’s integration of their pre-existing knowledge with initial patient information to form a representation of the problem, which is then used to guide further questioning to obtain more information and revise the problem representation [14]. This cycle is repeated until a final diagnosis is reached (diagnostic reasoning), which is later used to guide the management [14]. The process of clinical reasoning is conveyed to the listener or educator through the case presentation [10,11,15,16]. In our study, “Not knowing which information to include and which to omit” was the highest chosen reason by the students leading to challenging case presentations (Figure 1), which reflects the students’ struggle to elicit the relevant information from the patient’s story [2,10]. Results from our study would have been affected by different clinical reasoning skills and different levels of pre-existing knowledge that students possessed.

Onishi [15] has proposed a modification of the RIME model [12] and suggested the use of the vague, structured, organised, and pertinent (VSOP) model as a global rating scale for assessing clinical reasoning through case presentations. However, the model was designed and utilised for the outpatient setting, where patients are seen by a clinician for the first time. Our pilot study, on the other hand, took place in an inpatient cardiac surgery ward where most patients had already known their diagnosis, had spoken to multiple clinicians, and possibly have already had an operation. This may have affected the students’ clinical reasoning and case presentation, as they are used to following a structure for an undiagnosed patient.

Student’s self-assessment

Active engagement with the learning process and self-directed learning are pillars of the adult learning theory [17]. Early involvement of medical students in self-assessment will encourage involvement in learning and enhance continuous development as future doctors [18]. However, the validity and accuracy of self-assessments have been debated in the literature, with limited supporting evidence [19-22]. The Dunning-Kruger effect is a well-known cognitive bias where individuals with low ability tend to overestimate their competence, whereas highly skilled individuals tend to underestimate their competence [19,20]. In our study, students from group A evaluated their presentations (Figure 4) higher than students from group B in all aspects except one. This can be a demonstration of the Dunning-Kruger effect; as group B attended the tutorial session, they may have become more critical of their competence.

Around 80% of participants in group A were females compared to 55% in group B. In one study, female students tended to overestimate their competence [19], although this study did not look specifically into communication skills self-assessment. However, results from three meta-analyses reported that female students tend to underestimate their competence [22], while students in general tend to overestimate their competence when it comes to communication skills. No direct correlation could be established from our results.

Students from both groups reported difficulties in their consultations: 40% in group A and 35% in group B. As these reports are close, this factor would have had a trivial effect on the results of the self-assessments. Unfortunately, pre-teaching self-assessment questionnaires could not be collected from group B to compare it to post-teaching, and although students were asked to self-assess using the same criteria as their supervisors, different scales were used to answer the questions, which made comparison between the student’s self-assessment and the supervisor’s assessment challenging. Although students from group B were asked to evaluate their confidence before and after the tutorial session, different scales were used to answer the question. However, results demonstrated an increase in average confidence by 1.55 points on the Likert scale.

A gap in the current curriculum

The UOG curriculum covers communication skills teaching as part of the vocational and professional studies, which starts in the first year and continues throughout and mainly addresses communication skills with patients [23]. In addition, UOG offers a guide for both educators and students in clinical years, which suggests some techniques for case presentation such as SNAPPS and One-Minute Preceptor [9,24]; however, it does not offer much guidance on how to summarise a case. Unfortunately, the Clinical History and Examination Manual offered by the UOG only mentions that a summary of the case should be provided with no further elaboration on how to do that [25].

When looking at the assessment of a case summary and presentation, students are asked to hand over a patient to a colleague, refer a patient to a colleague, and present a patient review during rounds. All three tasks must be supervised by an educator as part of the Activities Log required in Senior Medicine Block. However, the Activities Log neither provides specific criteria to be met for achieving the task nor identifies the quality. The Activities Log is included within a document named Operation Colleague provided by UOG and can be accessed through The Clinical Years Booklet [24]. In the same block, students are assessed using an Objective Long Case Assessment [24], which includes an assessment of the clarity and accuracy of the student’s case summary. In our context, in the Cardiology and Cardiothoracic Block, students are only assessed using a Mini-Clinical Evaluation Exercise (Mini-CEX), which involves the assessor observing the student taking history and examining a patient, and a case-based discussion, which is a structured discussion of a clinical case the student was involved in. Unfortunately, neither form includes any assessment of the summary or presentation provided by the student [24].

Remarks on bedside teaching

Students’ free-text feedback highlighted that they appreciated the opportunity to take a history, perform an examination, and present the case back as one student per patient. The comments revealed that this was not the case in other settings, where one student takes history and another performs the examination. Clinical reasoning relies on both history-taking and physical examination to constitute a comprehensive clinical assessment in order to reach a diagnosis and formulate a management plan [26]. Therefore, maximum benefit from a teaching session will include a comprehensive consultation with the patient [27] rather than asking one student to take the history and another to examine the patient; however, due to time constraints, this may not always be feasible [1].

Limitations

Our supervisor’s assessment has demonstrated a small improvement in group B in comparison to group A. This could be attributed to the study design; no pre-teaching assessment was conducted for group B to compare it to post-teaching results. This was due to the limitations in our context: due to the length of the student placement and the number of CTFs, only one bedside teaching session could be offered for each student per block. Therefore, only one assessment could be conducted for each student. In addition, the tutorial session was delivered on the students’ first day of the placement, while the bedside sessions took place on different days over the whole period of the placement. As some students mentioned, they forgot to apply what was taught. This could have been avoided by providing the students with teaching materials in the form of a handout, a checklist, or the PowerPoint presentation (Microsoft Corporation) that was used for the tutorial.

Students attended the bedside session on different days; some of them were early in the placement, and others were towards the end. Any knowledge or experience gained throughout the placement that could have affected the results could not be excluded. Moreover, the supervisor’s assessment was conducted by three different CTFs. Each CTF assessed the student they were supervising during the bedside session, and although the assessment criteria were agreed upon by all the assessors, individual variations could not be excluded; therefore, assessment remained subjective.

Equally important, bedside sessions took place in a hospital ward, where patients could not be standardised. Students were exposed to a wide variety of patients and were assessed for different encounters. In addition, as students mentioned in their comments, some patients did not communicate clearly or were unsure of the timeline of their disease.

The small number of participants in our study is also another limitation to bear in mind. Further research with a larger number of participants would be beneficial.

## Conclusions

Our study identified a gap in our educational context, namely teaching and assessing verbal case summary and presentation, and outlined the possible reasons underpinning the problem. We aimed to address this gap, and a small improvement was achieved within the limitations. Teaching was well perceived by the students with the possibility of improving their communication skills and confidence. Students’ self-assessment demonstrated a Dunning-Kruger effect and was in keeping with the limited evidence supporting the accuracy of students’ self-assessment.

Further attention is required from curriculum designers, clinical educators, and assessors to bridge the gap in teaching and assessing verbal clinical case summary and presentation to undergraduate medical students.

## Appendices

Appendix A (Pre-teaching survey)

Demographics (For Research Purposes)

Age:

Sex:

Are you an international student?

Are you a postgraduate student?

Survey

Re: Giving a verbal case summary (brief presentation) of your consultation (history and examination) with a patient

How confident are you verbally summarising your consultation with a patient to your supervisor? (Very confident - Somewhat confident - Not confident - Not sure)

If not confident or not sure, why do you think it is challenging for you to verbally present a case summary? (select all that apply)

No previous teaching.

Not knowing what to include and what to omit.

Inability to put the history in chronological order.

Inability to summarise patient’s words using medical terms.

Not understanding what is going on with the patient.

Inadequate patient exposure/lack of opportunities to practise.

Have you received any previous specific teaching about case presentation/summary? (Yes - No - Not sure)

Appendix B (Lesson plan)

The lesson plan was designed using Gagne's model of instructional design [28] and was developed from literature review and assessors' personal professional experience.

**Table 6:** Lesson plan

Event and timing	Activity
Gaining attention (five to ten minutes)	1. Opening the PowerPoint presentation with the cover slide titled 'Case Summary, How to…?'. 2. Tutor introduction to the audience and asking students to fill out the pre-teaching survey.
Informing the learner of the objective (five minutes)	Learning objectives: 1. Describe the role of a case summary. 2. Give enhanced case summaries. 3. Compare a concise, clear case summary to a non-clear one. 4. Evaluate your own case summaries. 5. Evaluate your peers’ case summaries. In addition to setting the format of teaching, a large group tutorial, then small group bedside teaching sessions.
Stimulating recall of prerequisite learning (five to ten minutes)	The tutor asks the audience, "1. When is a case summary needed?" - The audience respond in OSCEs (objective structured clinical examinations), bedside teaching, ward rounds or in clinics. "2. And what does that mean?" – The tutor clarifies that it is a verbal communication between doctor and doctor; it is a summary of a long story and should demonstrate the presenter’s understanding of the patient's problem.
Presenting the stimulus material (five minutes)	The tutor begins to outline a format for delivering a case summary; it should be in chronological order, using medical terms, concise and clear with a conclusion that includes the diagnosis, any complications, and any treatments received.
Providing learning guidance (fifteen to twenty minutes)	The tutor provides multiple example scenarios explaining each of the requirements outlined beforehand. The tutor checks understanding several times and asks the audience if they have any questions.
Eliciting the performance (five minutes) (two and a half to three hours for each bedside session)	Students are offered some example scenarios and are asked to volunteer to deliver a case summary to the audience. Students later join a bedside teaching session in small groups of four or five students over the two-and-a-half-week placement. In the session, each student takes a history from a patient, performs an examination, and presents their case to their supervisor away from the patient.
Assessing the performance (five minutes)	The student’s presented summary is assessed using a supervisor’s assessment form (Appendix C). Each student evaluates their presentation using a student’s assessment form (Appendix D).
Providing feedback (five to ten minutes per student within the bedside session)	Debriefing and feedback are provided to the students in a safe, quiet environment away from the patients’ bedside.
Enhancing retention and transfer (five to ten minutes)	Through a student’s self-assessment and follow-up survey.

Appendix C (Supervisor’s assessment)

(1. Unsatisfactory - 2. Below average - 3. Average - 4. Very good - 5. Excellent)

Was the student’s summary:

Concise/omitting irrelevant information (1-2-3-4-5)

In chronological order (1-2-3-4-5)

In medical terms instead of patient’s words (1-2-3-4-5)

Clear (1-2-3-4-5)

Comprehensive (including disease, complication, treatment) (1-2-3-4-5)

Appendix D (Student’s self-assessment)

Re: your interaction today

Was your summary in chronological order? (Yes - No - Not sure)

Were you able to accurately summarise the patient’s wording using medical terms? (Yes - No - Not sure)

Was your summary concise (did you omit irrelevant information)? (Yes - No - Not sure)

Was your summary clear? (Yes - No - Not sure)

Was it comprehensive? (Yes - No - Not sure)

Did you encounter difficulties understanding what the patient was talking about, which in turn negatively affected your summary?

(Yes - No - Not sure)

Appendix E (Post-teaching survey)

Re: case summary teaching on your first day

On a scale from 1 to 5, 5 being the best

How useful was the teaching session? (1-2-3-4-5)

How relevant to your learning needs did you find it? (1-2-3-4-5)

How applicable was it to your practice today? (1-2-3-4-5)

How confident are you now in verbally summarising your consultation with a patient to your supervisor? (1-2-3-4-5)

Do you expect your verbal communication skills to improve? (1-2-3-4-5)

Is this a useful format of teaching? (1-2-3-4-5)

What did you find useful?

What could be improved?

Any comments?

Appendix F (Case summary teaching, follow-up survey)

Following the cardio block, what was your placement? (Neuro-Elective)

How would you rate your recollection of how to verbally present a case summary (teaching from Cardio placement)? (5 being the best) (1-2-3-4-5)

How would you rate your application of verbal presentation of a case summary (concise, chronological, clear, comprehensive) in the placement following Cardio? (5 being the best) (1-2-3-4-5)

If you rated the previous question low, what do you think was the reason? (I forgot how to verbally present a case summary - I forgot to apply it - I did not have a chance to verbally present a case - other)
